# The Malignant Transformation of Retrorectal Cystic Hamartomas With Blood Irregular Antibodies Positive: A Case Report

**DOI:** 10.1097/MD.0000000000002253

**Published:** 2015-12-11

**Authors:** Xiang-Rong Zhao, Chao Gao, Yong Zhang, Yong-Hua Yu

**Affiliations:** From the School of Medical and Life Sciences, Shandong Academy of Medical Sciences, Jinan University, Jinan, China (XRZ, CG); Department of Radiation Oncology II, Shandong Cancer Hospital and Institute, Jinan, China (XRZ, CG, YZ, YHY).

## Abstract

Retrorectal cystic hamartomas are rare congenital presacral lesions and malignancy is extremely rare. Although surgical excision is the essential for treatment, a unique feature of our case compared with previously reported tailgut cysts is that this patient's blood irregular antibodies are positive with higher operational risks.

A 44-year-old woman presented to our department complaining of pelvic and perineal pain for 6 months. Computed tomography (CT) scan of the abdomen and pelvis demonstrated a well-demarcated hypodense, multilocular cystic lesion, 10 cm in size, in the presacral region of the right of the midline. We found her blood irregular antibodies were positive in the preoperative examination. So she quitted surgery. Exploratory laparotomy and incision and drainage of pelvic tumor were operated. Postoperative routine pathology showed: (retroperitoneal tumors) moderately differentiated adenocarcinoma. Combined with clinical symptom and imaging, malignant transformation of retrorectal cystic hamartomas (tailgut cysts) was diagnosed. Taking into account that cyst is not sensitive to radiotherapy, so tumor necrosis factor (TNF) and raltitrexed were infused into the cysts and 3 cycles oxaliplatin (130 mg/m^2^) were completed. Now although the lesion is shrink, but yellow, viscous mucus still secrete constantly, 100 ml/w.

Given surgical excision is the essential for treatment, complete surgical excision should be implemented as far as possible. But if surgery cannot be carried out like the presented case, systemic chemotherapy and local radiotherapy are also available, which can alleviate the symptoms of oppression and improve the quality of life partly.

## INTRODUCTION

Tailgut cysts or retrorectal cystic hamartomas are rare congenital presacral lesions identified in all age groups. They are believed to arise from the remnants of the embryonic hindgut. Retrorectal cystic hamartomas are 3 times more common in women than men. They can be detected at any age, including infancy.^1,2^ Malignancy in tailgut cysts is extremely rare, the majority being adenocarcinomas and carcinoid tumors.^3–5^ We report a case of adenocarcinomas associated with a tailgut cyst. A unique feature of our case compared with previously reported tailgut cysts is that this patient's blood irregular antibodies are positive with higher operational risks.

## CASE PRESENTATION

**FIGURE 1:**
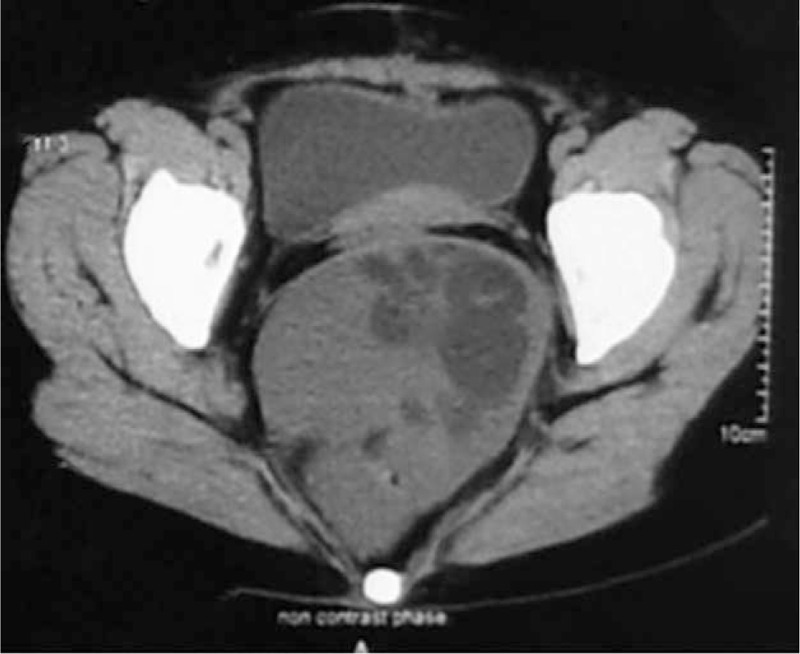
The CT image took in February 2013, 10 cm in size, in the presacral region to the right of the midline.

**FIGURE 2:**
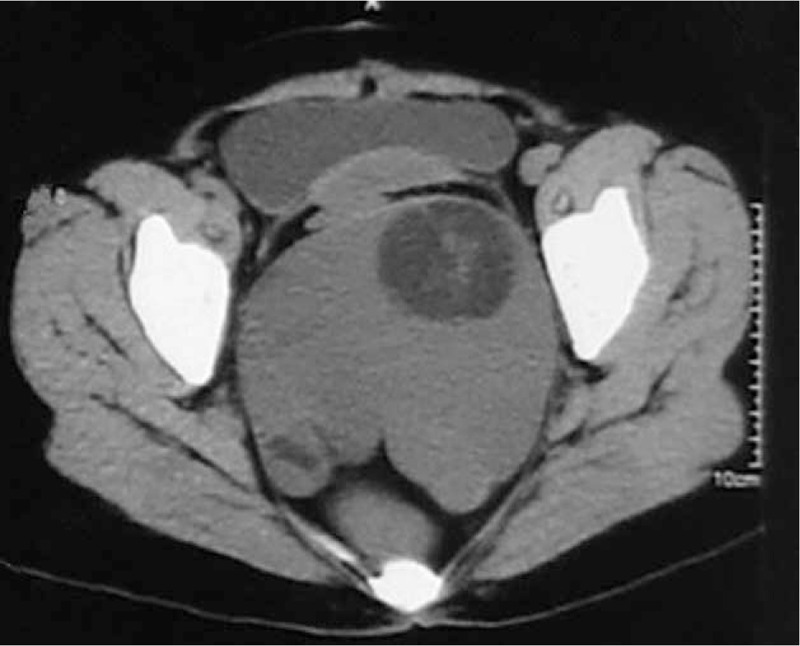
The CT image took in October 2014, 14 cm in size.

**FIGURE 3:**
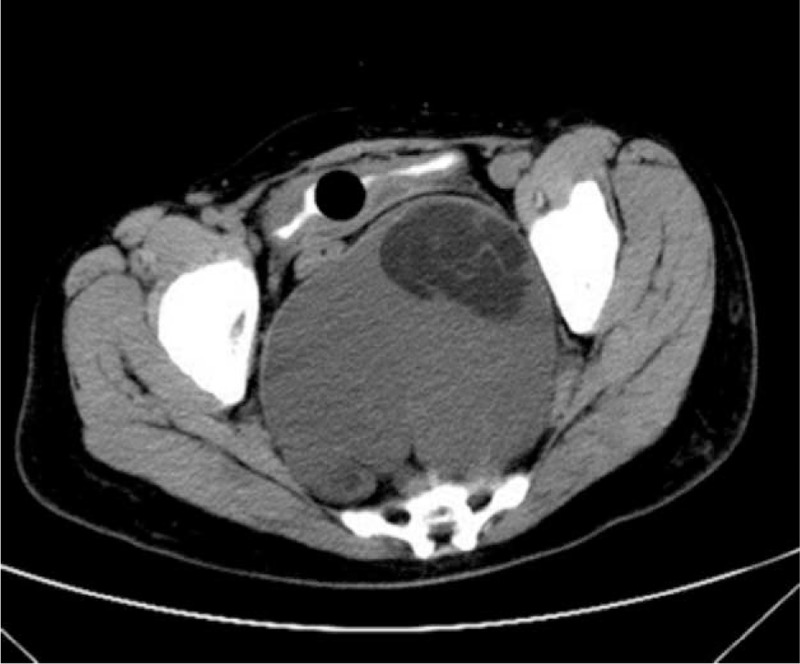
The CT image took in January 2015, 16 cm in size.

**FIGURE 4:**
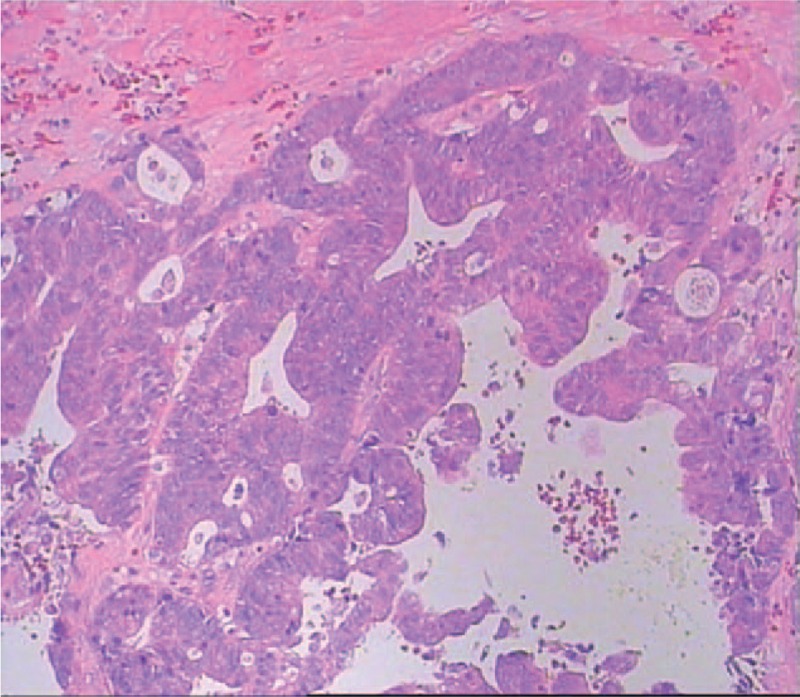
Postoperative routine pathology: Combined with clinical symptom and imaging, malignant transformation of retrorectal cystic hamartomas (tailgut cysts) was diagnosed.

A 44-year-old woman presented to our department complaining of pelvic and perineal pain for 6 months. We found no abnormality on physical examination in February 2013. A nontender, extrinsic, well-defined presacral mass was discovered by digital rectal examination which compressed the rectum. No mucosal abnormalities were revealed in the sigmoidoscopy. Routine laboratory tests and tumor marker results were within normal limits. Computed tomography (CT) scan of the abdomen and pelvis demonstrated a well-demarcated hypodense, multilocular cystic lesion, 10 cm in size, in the presacral region of the right of the midline (Fig. 1). We found her blood irregular antibodies were positive in the preoperative examination. So there was little chance to finish cross matched blood. It was full of hazard to hemorrhage as the lesion was huge. So she quitted surgery. At exploratory laparotomy for excision of the lesion, we found that the mass was adherent to and not easily separated from the rectum and surrounding pelvic wall. The size of the mass had slight change until October 2014. Abdominal CT demonstrated that lesion was bigger than the last CT image, 14 cm in size (Fig. 2). What's more, the patient had difficulty in passing her motions with shape changing. But the patient still refused to receive treatment. In January 2015, the patient experienced ventosity and progressive aggravation. Abdominal CT demonstrated that lesion was bigger than the last CT image, 16 cm in size (Fig. 3). Taking into account the cystic mass, paracentesis was carried out with about 2000 ml yellow liquid extracted. Cancer cells were not found in cytological tests. Abdominal CT demonstrated that lesion shrank. In March 2015, laboratory test showed carcinoembryonic antigen (CEA) elevated. We realized the possibility of malignant transformation. So exploratory laparotomy and incision and drainage of pelvic tumor were operated. We found that the mass was adherent to and not easily separated from the rectum and surrounding pelvic wall. Adipose and osseous tissues were seen in the cystic lesion. Part of the lesion was resected with a drainage tube indwelled. Postoperative routine pathology showed: (retroperitoneal tumors) moderately differentiated adenocarcinoma. Combined with clinical symptom and imaging, malignant transformation of retrorectal cystic hamartomas (tailgut cysts) was diagnosed (Fig. 4). Then tumor necrosis factor (TNF) and raltitrexed were infused into the cysts and 3 cycles oxaliplatin (130 mg/m^2^) were completed. Now although the lesion is shrinking, but yellow, viscous mucus still secrete constantly, 100 ml/w. As surgical excision is the essential for treatment, we still suggest this patient to operate by prestoring herself blood and autologous blood transfusion.

This study was approved by the institutional review boards and ethics committees of Shandong Cancer Hospital and Institute.

The patient has consented for the publication of the present case report.

## DISCUSSION

In the literature, various terms have been used to describe tailgut cysts, including cyst of the postanal intestine, mucus-secreting cyst, retrorectal cystic hamartomas, simple cyst, retrorectal cyst, and enterogenous cyst.^1,3,6^ They are believed to arise from the remnants of the embryonic hindgut. The human embryo possesses a true tail as an extension of the primitive gut. A tailgut cyst is a rare congenital lesion representing a nonregressed tail. It was most often found in asymptomatic middle-aged women.^1^

Because the lesion is located in the bathy-pelvis and extremely rare, so it is difficult to diagnosis. Imaging examination is an effective way to diagnosis. Ultrasound shows multilocular cystic lesions with internal echoes due to mucoid material or inflammatory debris. Abdominal CT demonstrated a well-demarcated hypodense, bilocular cystic lesion in the presacral region. Some may have calcifications. There is one report of endoscopic ultrasonography-fine needle aspiration (EUS-FNA) with a flexible echoendoscope.^7^ Puncture should be performed when other etiologies are considered or if malignant degeneration changes management. It is the most accurate diagnosis. What's more, malignant transformation should be paid to special attention. It can be used as a marker of malignant transformation that CEA elevated.

Malignant transformation in the form of adenocarcinoma or neuroendocrine tumors, although rare, does occur. Mathis et al^8^ reported 31 patients with tailgut cysts and 4 of them (13%) had malignant findings: adenocarcinoma in 3 and neuroendocrine tumors in 1. They concluded that presacral tailgut cysts should be removed because of the risk of malignant transformation, because 2 of the 4 patients with malignancy had died through the follow-up period. Therefore, complete resection of these lesions is recommended, as surgical excision is the gold-standard treatment.^9^ But simple cyst excision or drainage leads to recurrence and possible infection. The treatment of choice is complete excision of the lesion, with resection of the coccyx and a surrounding margin of grossly normal tissue.^3,10^ If surgery cannot be carried out, systemic chemotherapy and local radiotherapy can be implemented.

Prognosis for tailgut cysts with malignant transformation depends on the status of complete surgical resection and tumor histology and grade. Local recurrences and distant metastasis have been occasionally reported. Two cases with local recurrences and 1 case with a distant metastasis have been reported in adenocarcinomas arising in tailgut cysts in the literature.^11,12^

## CONCLUSION

Tailgut cysts should be taken into account, if a retrorectal mass cannot be diagnosis clearly. Given surgical excision is the essential for treatment, complete surgical excision should be implemented as far as possible. Malignant transformation, although unusual, may be focal, so meticulous examination of the gross mass and thorough samplings are important. The coccyx and a surrounding margin of grossly normal tissue should also be excision, when malignant transformation was confirmed. But if surgery cannot be carried out like the presented case, systemic chemotherapy and local radiotherapy are also available, which can alleviate the symptoms of oppression and improve the quality of life partly.
