# The malignant transformation of retrorectal cystic hamartomas with blood irregular antibodies positive: A case report: Erratum

**DOI:** 10.1097/MD.0000000000006010

**Published:** 2017-01-20

In the article “The malignant transformation of retrorectal cystic hamartomas with blood irregular antibodies positive: A case report”,^[1]^ which appeared in Volume 94, Issue 49 of *Medicine*, Dr. Zhao's affiliations were originally given incorrectly. The correct affiliations are as follows:

1. School of Medicine and Life Sciences, University of Jinan-Shandong Academy of Medical Sciences, Jinan, China and
2. Department of Radiation Oncology, Shandong Cancer Hospital and Institute, Shandong Academy of Medical Sciences, Jinan, China.

All other affiliations appeared correctly in the original article.
